# The role of Cysteine 6.47 in class A GPCRs

**DOI:** 10.1186/1472-6807-13-3

**Published:** 2013-03-15

**Authors:** Mireia Olivella, Gianluigi Caltabiano, Arnau Cordomí

**Affiliations:** 1 Departament de Biologia de Sistemes, Universitat de Vic, Vic, Barcelona 08500, Catalonia; 2 Laboratori de Medicina Computacional, Unitat de Bioestadística, Facultat de Medicina, Universitat Autònoma de Barcelona, Bellaterra, Barcelona 08193, Catalunya

## Abstract

**Background:**

The CWxP motif of transmembrane helix 6 (x: any residue) is highly conserved in class A GPCRs. Within this motif, W6.48 is a big star in the theory of the global “toggle switch” because of its key role in the activation mechanism of GPCRs upon ligand binding. With all footlights focused on W6.48, the reason why the preceding residue, C6.47, is largely conserved is still unknown. The present study is aimed to fill up this lack of knowledge by characterizing the role of C6.47 of the CWxP motif.

**Results:**

A complete analysis of available crystal structures has been made alongside with molecular dynamics simulations of model peptides to explore a possible structural role for C6.47.

**Conclusions:**

We conclude that C6.47 does not modulate the conformation of the TM6 proline kink and propose that C6.47 participates in the rearrangement of the TM6 and TM7 interface accompanying activation.

## Background

G protein-coupled receptors (GPCRs) are versatile signaling molecules that regulate a vast amount of cellular processes responding to hormones and neurotransmitters [1]. They are present in almost every eukaryotic organism, including fungi and plants, and they are highly diversified in mammalian genomes, representing 2-3% of the human proteome [2]. GPCRs transduce external signals as diverse as photons, odors, pheromones, biogenic amines, neuro-peptides, proteases, glycoprotein hormones and ions (among others) into the cell interior. The response is operated through second messenger cascades controlled by both heterotrimeric guanine nucleotide-binding proteins (G-proteins) [3] or G protein-independent pathways [4]. GPCRs constitute one of the most important pharmaceutical targets, with 30% of marketed drugs acting through them [5]. Mammalian GPCRs are classified in three major families or classes (A, B and C) from which class A is the largest and most studied [6]. Although each GPCR plays a single role in mediating physiological response, many conserved sequential and structural features are encountered among them, mainly near the cytoplasmic part where receptors bind to the G protein [7,8]. Towards the extracellular side, divergence increases [9,10] because of the wide diversity of ligands that need to be bound.

The relatively large repertoire of class A GPCR crystal structures currently available [11-29] (including complexes with agonists, antagonists and inverse agonists) shed light into the understanding of GPCRs’ molecular architecture, as well as on the events responsible for signal transmission (see Table 1). Receptors are thought to be in equilibrium among different states, being inactive ones the most populated in absence of ligand. Agonist binding alters the energetic balance towards actives states triggering activation responses. This implies changes in the conformation of a few residues that are responsible for the formation and disruption of hydrophobic and hydrogen-bond interactions between specific groups of residues. Most of the highly conserved residues often belong to “micro-switches” that swap between different conformations, and constitute an extended allosteric interface between transmembrane (TM) domains [30]. These micro-switches span from the extracellular part to the intracellular G-protein binding site and regulate the equilibrium between inactive and active conformations. This may trigger, eventually, global movements connecting ligand binding with intracellular signaling. R3.50 and D3.49 (of the DRY motif; Ballesteros and Weinstein numbering scheme will be used through this text [31]), Y5.58, Y7.53 (of the NPxxY motif), and W6.48 (of the CWxP motif), are some of the most studied micro-switches [32-36].

**Table 1:** The 6.47-7.45 interaction in the analyzed crystal structures

	**Crystal structure**			**TM6-TM7 hydrogen bond**			
**Receptor name**	**Structure name**	**PDB ID**	**Res.(Å)**	**Pair**	**N7.45**	**D… A (Å)**	**Cβ-D…A (°)**
β2-adrenergic	β_2_AR^INV^	2RH1 [11]	2.4	C6.47-N7.45	CO	3.8	84.5
	β_2_AR^AGO^	3SN6 [12]	3.2		CO	4.4	81.2
β1-adrenergic (turkey)	β_1_AR^INV^	2VT4 [13]	2.7	C6.47-N7.45	CO	3.7	79.3
	β_1_AR^AGO^	2Y02 [14]	2.6		CO	3.9	85.3
Dopamine D3	D3R^INV^	3PBL [15]	2.9	C6.47-N7.45	CO	3.8	81.6
Histamine H1	H1R^INV^	3RZE [16]	3.1	C6.47-N7.45	CO	4.2	80.9
Adenosine A2A	A_2A_R^INV^	4EIY [17]	1.8	C6.47-N7.45	NH_2_	3.9	84.5
	A_2A_R^AGO^	2YDV [18]	2.6		CO	4.3	74.4
κ opioid	κOR^INV^	4DJH [19]	2.9	C6.47-N7.45	CO	4.1	81.2
μ opioid (mouse)	μOR^INV^	4DKL [20]	2.8	C6.47-N7.45	CO	3.6	87.5
δ opioid (mouse)	δOR^INV^	4EJ4 [21]	3.4	C6.47-N7.45	NH_2_	3.8	89.8
N/OFQ Opioid	N/OFQ^INV^	4EA3 [22]	3.0	C6.47-N7.45	NH_2_	4.2	85.6
Sphingosine 1-phosph. 1	S1PR_1_^INV^	3V2Y [23]	2.8	C6.47-N7.45	NH_2_	3.4	81.1
CXCR4 chemokine	CXCR4^INV^	3ODU [24]	2.5	C6.47-H7.45	-	-	-
M2 musc. ach.	M2R^INV^	3UON [25]	3.0	T6.47-N7.45	NH_2_	3.2	120.2
M3 musc. ach. (rat)	M3R^INV^	4DAJ [26]	3.4	T6.47-N7.45	NH_2_	4	140.7
Rhodopsin (bovine)	bRho^INV^	1GZM [27]	2.7	C6.47-T7.44	-	3.7	101.8
	bRho^AGO^	3PQR [28]	2.9		-	8.8	-
Rhodopsin (squid)	sRho^INV^	2Z73 [29]	2.5	Q6.43-S7.54	-	2.4	111.2

(Left) Crystal structures used within the manuscript (INV: inverse agonist or antagonist; AGO: agonist); Protein Data Bank Identification Code and Resolution; receptor structures are human unless stated. (Right) Characterization of the TM6-TM7 hydrogen bond: pair involved, N7.45 rotamer (CO/NH_2_ indicates the closer group to C6.47 as in their crystal structure), donor(D)-acceptor(A) distance and Cβ-donor(D)···acceptor(A) angle.

The CWxP motif in TM6 (6.47-6.50) is highly conserved in non-olfactory class A GPCRs, with C6.47, W6.48 and P6.50 present in 71%, 78% and 98% of sequences, respectively (see Methods). P6.50 creates a huge kink in TM6 helix (causing a helix bend of around 35°), far bigger than a usual Proline in a transmembrane helix (around 20°) [37]. Because an outward movement of the cytoplasmic side of TM6 is known to accompany receptor activation, it was initially proposed that the Pro-kinked helix could act as a mechanical hinge [38,39]. In fact, these pioneering experiments and computer simulations on rhodopsin originated one of the central paradigms in GPCR activation: the so-called “rotamer toggle switch”. This consisted in a concerted change of C6.47, W6.48 and F6.52 side-chain rotamers between inactive and active states, which were thought to originate the opening of TM6 [40]. In specific, transition of W6.48 from *gauche+* to *trans* and of C6.47 from *trans* to *gauche+* was proposed by Shi and collaborators [38,39]. The low resolution structure of metarhodopsin I, determined by electron crystallography [41], supported the rotamer change of W6.48 as did more recent infrared spectroscopy experiments of rhodopsin photocycle [42]. However, and quite-unexpectedly, none of the available pairs of inactive-active structure are consistent with these rotameric changes (see Table 1 and references therein), questioning the rotamer toggle switch model. Still, it is known from mutagenesis experiments that W6.48 is essential for signaling of some (but not all) receptors [40,43]. On the other hand, there is no current explanation for the large conservation of C6.47 and for the effects on activity reported by various C6.47 mutants. In particular, C6.47T in β_2_-adrenergic receptor [39] and C6.47R in thyrotropin hormone receptor [44], both led to constitutive Gα_s_ activation. Similarly, C6.47 mutants in cannabinoid receptors CB_1_ and CB_2_ modified both ligand recognition and receptor activation [45,46].

The present study reports an extensive analysis of the available GPCRs crystal structures combined with results from molecular dynamics (MD) simulations of model peptides in a membrane mimic aimed at characterizing the role of C6.47. Our results support that C6.47 is a key element of a conserved micro-switch within the TM6/TM7 interface. We show that Cys side-chain possesses unique physicochemical properties and rotameric preferences (compared to Ser and Thr) that allows formation of specific interactions not possible for other small hydrogen-bonding capable residues.

## Results and discussion

### Residue prevalence and side chain conformation of position 6.47

70% of class-A GPCRs have Cys at position 6.47, whereas the homologous Ser and Thr account, respectively, for only 10% and 4% of the sequences (see Methods). Accordingly, most presently available crystal structures of GPCRs contain cysteine at this position except for muscarinic receptors M2R and M3R, which feature Thr, and for squid rhodopsin, which features Ser (see Table 2). This suggests that a small residue with hydrogen-bonding capabilities is required at position 6.47, but also a preference for Cys rather than Ser or Thr. Structural analysis shows that, among receptors containing C6.47, the residue is in *gauche +* conformation (*g+*) in all cases except rhodopsin (*trans*, t). M2R^INV^ and M3R^INV^ feature T6.47 in *gauche-* (*g-*) and sRho^INV^ has S6.47 in *trans* conformation. For those receptors in which both agonist- and inverse agonist-bound structures are available (β_2_AR^INV^-β_2_AR^AGO^, β_1_AR^INV^-β_1_AR^AGO^, A_2A_R^INV^-A_2A_R^AGO^, bRho^INV^-bRho^AGO^) there is no change in 6.47 rotamer conformation. Thus, crystal structures do not support the rotamer change for residue 6.47 accompanying receptors activation as hypothesized by Shi et al [38,39].

**Table 2:** Residues present at positions 6.47 and 7.42-7.48

**Receptor**	**Position**							
	**6.47**	**7.42**	**7.43**	**7.44**	**7.45**	**7.46**	**7.47**	**7.48**
β2-adrenergic	**C*g+***	G	Y	V	**N**	S	G	F
β1-adrenergic (turkey)	**C*g+***	G	Y	V	**N**	S	A	F
Dopamine D3	**C*g+***	G	Y	V	N	S	A	L
Histamine H1	**C*g+***	G	Y	I	**N**	S	T	L
Adenosine A2A	**C*g+***	S	H	**T *g+ /t***	**N**	S	C	L
κ opioid	**C*g+***	G	Y	T	**N**	S	S	L
μ opioid (mouse)	**C*g+***	G	Y	T	**N**	S	C	L
δ opioid (mouse)	**C*g+***	G	Y	A	**N**	S	S	L
N/OFQ Opioid	**C*g+***	G	Y	V	N	S	C	L
Sphingosine 1-phosph. 1	**C*g+***	A	V	L	**N**	S	G	T
CXCR4 chemokine	**C*g+***	A	F	F	**H**	C	A	L
M2 musc. ach.	**Tg-**	C	Y	I	**N**	S	T	I
M3 musc. ach. (rat)	**Tg-**	C	Y	I	**N**	S	T	V
Rhodopsin (bovine)	**Ct**	A	K	**T *g+ */t**	Sg-	A	V	Y
Rhodopsin (squid)	**S*g+***	A	K	A	**St**	A	I	H

Rotamer of C, S and T (g+/-: gauche+/-, t: trans) is indicated when relevant; residues involved in TM6-TM7 hydrogen bonds are displayed in bold.

### MD simulations of A/C/T/SxxP model peptides

**Figure 1:**
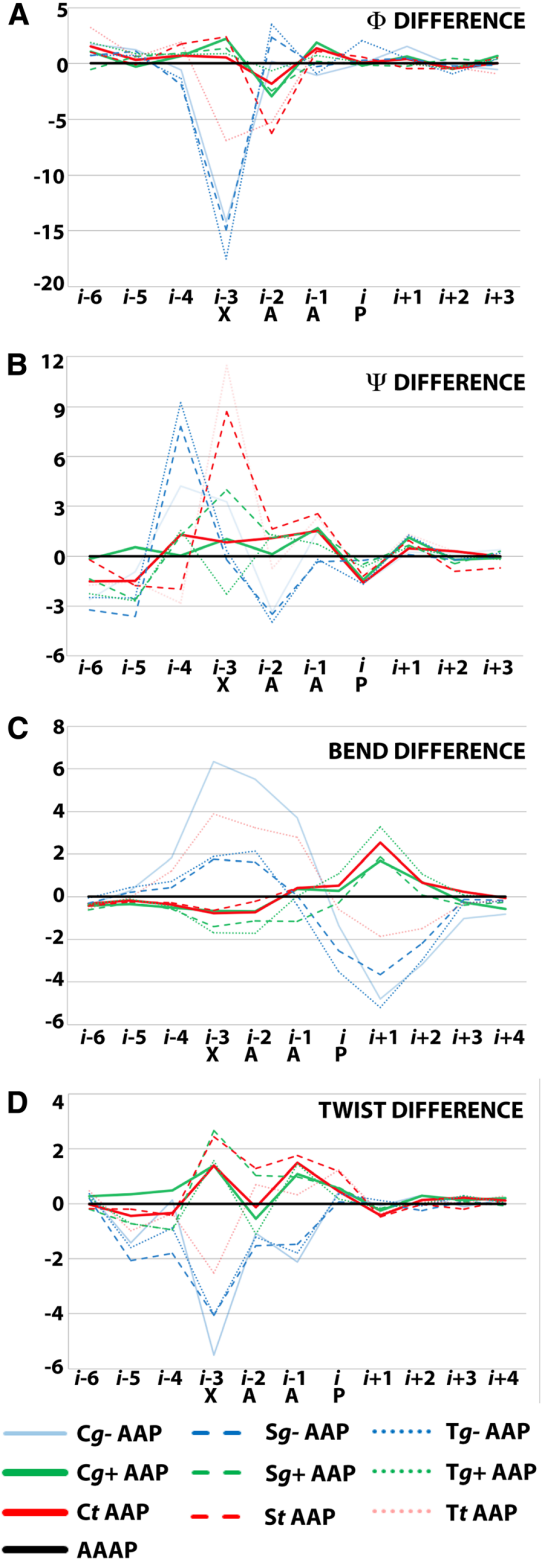
**Analysis of TM6 irregularities computed from MD simulations of polyalanine peptides containing the CAAP, SAAP, TAAP motifs in the gauche-, gauche + and trans conformers.** Difference between average Φ (**A**) and Ψ (**B**) dihedral angles, unit bend (**C**) and unit twist (**D**) profiles. CtAAP and Cg+AAP are the unique peptide conformations that present very similar helical geometrical parameters to AAAP. Sg+AAP peptide present certain distortion relative to AAAP peptide and Cg-AAP, Sg-AAP, StAAP, Tg+AAP, Tg-AAP, TtAAP present the most distorted helical geometrical parameters relative to AAAP peptide. Bend and twist angles assigned at position i corresponds to the value computed for (*i-3*, *i*) and (*i*, *i + 3*) and for (*i-3*, *i*), respectively.

We have previously shown how specific Ser and Thr conformers can efficiently modulate the structure of a neighboring proline-kink by forming hydrogen bonds with the backbone carbonyl at positions *i-3* or *i-4* (with respect to proline, *i*) [37,47,48]. In the present study, we performed MD simulations of model peptides containing Ser, Thr and Cys at position *i-4* with respect to proline, in a hydrophobic solvent (see Method). Analogous peptides containing C/T/SWAP motifs exhibit a similar profile (see Additional file 1: Figure S1). The aim of these simulations was to establish whether or not Cys could alter the structure of a regular proline kink and quantify the extent of the alteration compared to Ser and Thr [49]. Figure 1 shows the change in *Φ* and *Ψ* angles and in *local twists* and *bends* for peptides with respect to the reference AAAP peptide, for C/T/SAAP motifs with the C/T/S rotamer fixed to *g+, g-* or *t* (see Methods). In terms of Φ and Ψ, it can be seen that all *g-* conformers induce a ~15° decrease in Φ angle profile (relative to the WT) at position *i*-3, and an increase (8-9° for Ser and Thr, 4° for Cys) in Ψ angle profile at position *i-4*. *Trans* conformers of Ser and Thr (but not of Cys) induce a smaller but parallel effect: ~6° decrease in the Φ angle at position *i-2* (and also at position *i-3* for Thr) and ~10° increase in Ψ angle profile at *i-3* position. Regarding bend profiles, all of them exhibit a bimodal shape, where Cys g+, Cys t and Ser *g+* exhibit the smallest deviations (<2°) relative to the WT. Finally, twist angle in all g- conformers exhibits a pronounced decrease in the region between *i-6* and *i*. Collectively, it can be concluded that C*g-* (blue continuous line) and all rotamers of Ser and Thr (broken and dotted lines) modify the standard proline kink profile (black line). Opposite, profiles of both *Cg+* and *Ct* reveal no important alterations of the standard Pro-kink (continuous lines). Taking in consideration that C*g-* rotamer is forbidden because of steric clash [47] (see Table 3), cysteine turns out to be the only small hydrogen-bonding capable residue that does not modify the profile of a standard Pro-kink in its allowed conformations. On the other hand, Ser and Thr would require further adaptation of the environment in order to be tolerated at position 6.47.

**Table 3:** Population of C/S/T rotamers in transmembrane helices from crystal structures

**Residue**	**Conformation (%)**		
	***gauche-***	***gauche+***	**trans**
Cysteine	0	71	29
Threonine	15	84	1
Serine	20	52	28

Data arranged from reference [47].

### TM6/TM7 interactions involving 6.47

**Figure 2:**
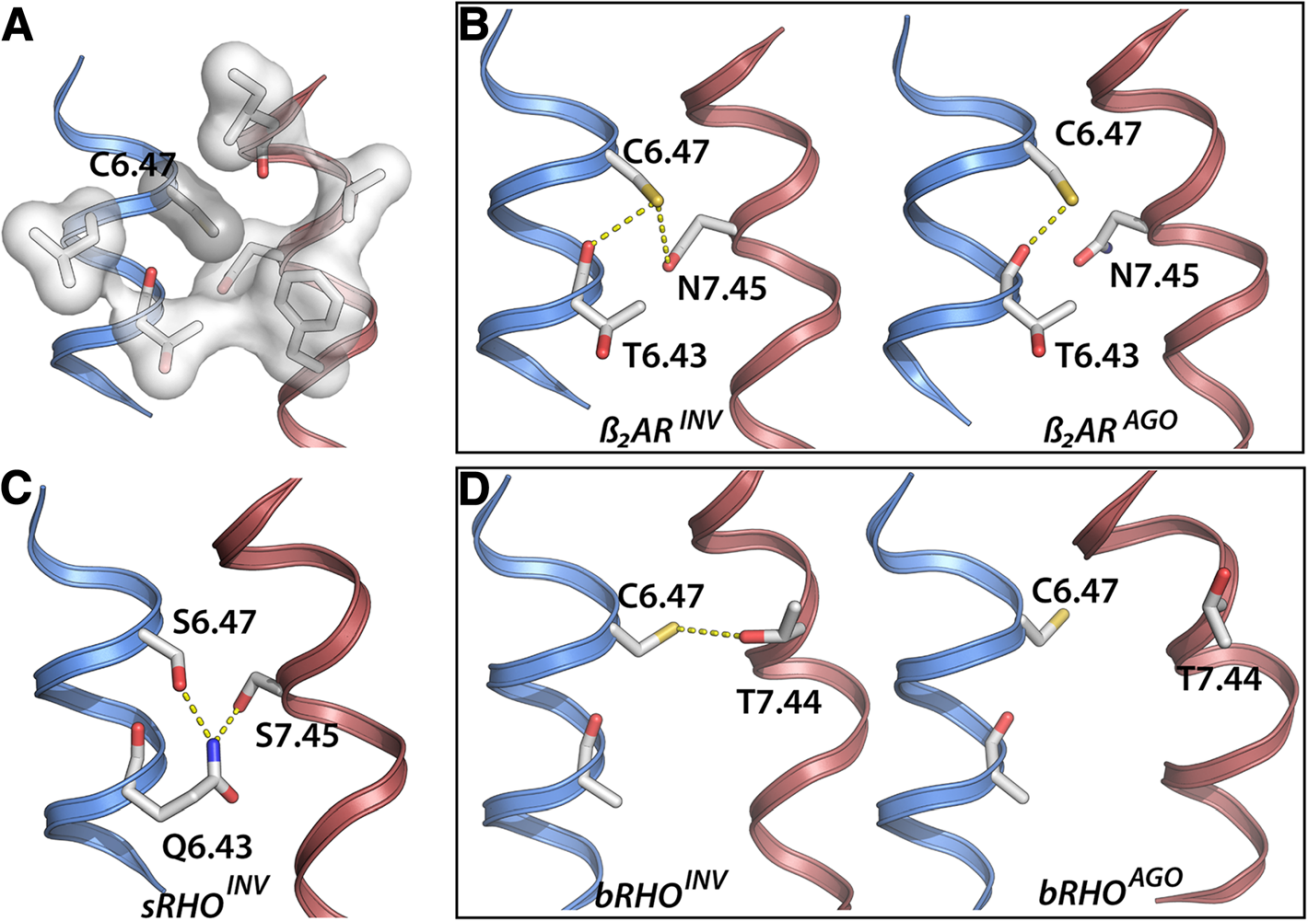
**The TM6-TM7 interface in β**_**2**_**AR**^**INV **^**structure. A**) Hydrophobic residues (white surface) and free carbonyl groups (red) surrounding C6.47 (grey surface). **B**-**D**) Hydrogen bond network at the TM6 and TM7 interface (indicated by dotted lines). **B**) In β_2_AR^INV^ (left) C6.47 forms a hydrogen bond with N7.45 and eventually with the backbone of 6.43; in β_2_AR^AGO^ (right) the hydrogen bond between 6.47 and N7.45 is broken and C6.47 interacts only with the backbone of 6.43. **C**) sRHO^INV^ features Q6.43 bridging S6.47 and S7.45. **D**) In RHO^INV^ (left) C6.47 is interacting with T7.44 *g+*; in RHO^AGO^ the hydrogen bond between Cys 6.47 and T7.44 has broken. Color code is as follows: TM6 (blue), TM7 (pale red).

Having discarded C6.47 as a modulator of TM6 kink, it is then likely that its high conservation is due to specific interactions played by the thiol group. Crystal structures show that the central segments of both TM6 and TM7 are in intimate contact. The maximum proximity is found between residues 6.47 and 7.42 (~3.5 Å within the closest atoms). Position 6.47 is precisely the hinge of the rigid body movement of the cytoplasmic side of TM6 associated to GPCR activation [28,50,51]. Figure 2A shows a detailed view centered at C6.47 for β_2_AR^INV^ structure (PDB:2RH1) taken as representative. It can be seen that the thiol group is surrounded by three aliphatic residues (T6.43, L6.46, V7.44), one aromatic residue (F6.48), but also by hydrogen bond-capable groups (backbone carbonyl groups of residues 6.43 and 7.41 and the side chain of N7.45). This dual nature at the environment of 6.47 is common to the remaining structures and perfectly suits the physiochemical properties of Cys thiol group. Analysis of proteins of known structures show that cysteine environment are closer to those of Ile methyl groups than to Ser [52]. On the other hand, Cys is a moderately good hydrogen-bond donor as demonstrated by the fact that more than two thirds of the thiol groups in the Protein Data Bank or the Cambridge Structural Database co-occur with suitable acceptors, manly carbonyl groups [53-57]. Cysteine has also been described to participate in thiol- aromatic π-type hydrogen bonds [58].

Based on molecular modeling and site-directed mutagenesis studies, a C6.47-N7.45 hydrogen bond interaction has recently been proposed in the gonadotropin-releasing hormone receptor [59]. Since all receptors with available structures feature N7.45 (66% conserved) except bRho/sRho (contain S:12%) and CXCR4 (contains H:8%) we assessed, by conducting systematic analysis on the available crystal structures, if this interaction was likely to exist in other GPCRs.

Available crystal structures differ in the assignment of CO and NH_2_ groups for N7.45 (see Table 1). This it is not surprising because even at 1.8 Å resolution (the highest obtained for a GPCR structure; see Table 1) it is not possible to correctly assign all side chain rotamers. Since it is unlikely that the orientation of the CO/NH_2_ groups of N7.45 is not the same in all receptors (this position is surrounded by many conserved residues) we evaluated the two possible orientations using NQ-Flipper (see Methods) [60]. The results revealed that the conformer present in β_2_AR^INV^ (PDB:2RH1) is the most favorable in all structures. We, therefore, performed our analysis assuming the rotamer present in β_2_AR^INV^ as the correct one. Table 1 displays S(Cys6.47) · · · O(Asn7.45) distances and Cβ-S · · · O angles measured on the crystal structures of Asn7.45-containing receptors (see Methods). In all structures containing C6.47 except β_2_AR^AGO^ structure (3SN6), geometries are compatible with the existence of a hydrogen bond between C6.47 and N7.45 [53-57]. It should be noted that sulfur hydrogen bond has a strong dispersion energy component [61] and, therefore, the directionality of the hydrogen bond is less pronounced than in conventional hydrogen bonds, leading to flat potential energy surfaces that are attractive even at large distances [54,62].

Figure 2B shows a comparison between β_2_AR^INV^ and β_2_AR^AGO^, displaying the breakage of the C6.47 · · · N7.45 hydrogen bond upon agonist binding. In parallel, it can be seen in the figure that carbonyl group of 6.43 in β_2_AR^INV^ is oriented toward the amide group of C6.47, whereas in β_2_AR^AGO^ it points towards thiol C6.47 group.

As a result, there is a decrease in the Cys6.47(S) · · · 6.43(backbone CO) distance, which suggests the stabilization of an alternative hydrogen bond in β_2_AR^AGO^. This leads us to propose that the breakage of the C6.47-N7.45 hydrogen bond is related to activation in β_2_AR. For A_2A_R the trend of that withdrawnness of N7.45 from C6.47 is clearly maintained. The fact that β_1_AR^AGO^ and A_2A_R^AGO^ structures reflect a smaller change as compared to β_2_AR^AGO^ could be due to the fact that the structures do not account for fully active-like states, as it has previously been proposed [18,63]. Possible reasons could be the lack of TM6 opening (maybe because of absence of G-protein) or, in the case of β_1_AR^AGO^, the presence of thermostabilizing mutations (including F7.48M contacting C6.47). On the other hand, H1R^INV^, known for its high basal activity, exhibits a distance in between β_2_AR^INV^ and β_2_AR^AGO^ even in presence of antagonist [16].

Muscarinic receptors are of the few GPCRs that present a Thr in position 6.47. Despite that M2R^INV^ and M3R^INV^ exhibit the analogous hydrogen bond interaction between T6.47 and N7.45. The rotamer for Thr is *g-*, despite *g*+ is the most populated rotamer for Thr (see Table 2). This anomaly seems to originate from the steric clash that Thr methyl group would give with TM7 in the latter conformer. Rhodopsins belong to the small group of receptors that contain S7.45. Because serine is too short to reach the side-chain of 6.47, rhodopsins seem to have adopted alternative mechanisms to control TM6-TM7 interface. This could be related with the fact that retinal is attached to the neighboring K7.43. Figure 2C displays the structure of sRHO^INV^ that, oddly, features S6.47 in the *g*+ rotamer conformation. It can be seen that the receptor contains residue Q6.43 that bridges S6.47 and S7.45. In the case of bRho (Figure 2D), bRho^INV^ contains an alternative TM6-TM7 hydrogen bond between C6.47 and T7.44. Comparison between bRho^INV^ and bRho^AGO^ reveals that in the former, *trans* C6.47 forms a hydrogen with *g-* T7.44, whereas in the latter, T7.44 has changed conformation to *trans*, breaking C6.47-T7.44 interaction (see Figure 2D). Thus, as in β_2_AR^INV^/β_2_AR^AGO^ pair, there is a breakage of the C6.47-TM7 hydrogen bond associated to activation.

CXCR_4_^INV^ is the only resolved receptor whose sequence contains H7.45 (instead of Asn). Although histidine is 87% conserved in chemokine receptors, CXCR4^INV^ shows no (direct) interaction between 6.47 and H7.45.

### 6.47 modulates D2.50-N7.49 interaction

**Figure 3:**
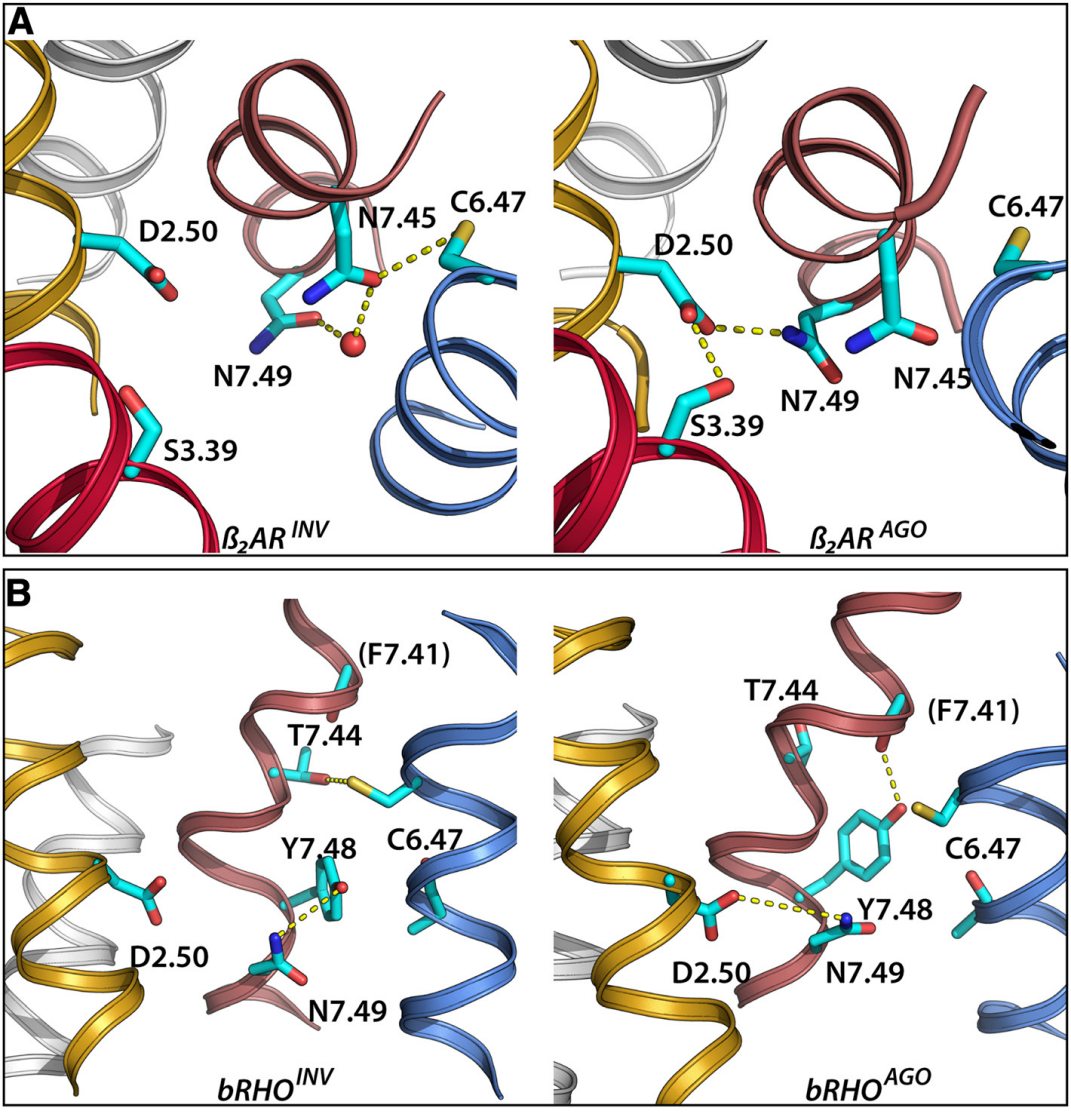
**Hydrogen bond network associated to C6.47 in both inverse-agonist (left) and agonist (right) bound structures of β**_**2**_**AR (A) and bRho (B).** Panel **A**: C6.47 hydrogen bonds N7.45 which retains N7.49, avoiding interaction of the latter with D2.50 (left). Breaking of C6.47-N7.45 interaction releases (right) N7.49 permitting its interaction with D2.50. Panel **B**: the release of both C6.47-T7.44 and Y7.48-N7.49 hydrogen bonds (left), permits N7.49 to form a hydrogen bond with D2.50. Helices are colored as follows: TM1:white, TM2:gold, TM3:red, TM6:blue and TM7:pale-red. The remaining helices have been omitted for better clarity. Relevant side-chains are shown as sticks (cyan) and hydrogen bonds are indicated by dashed lines. A crystallographic water molecule is shown as spheres.

In β_2_AR^INV^ N7.45 and N7.49 side chains form a water-mediated hydrogen bond. In β_2_AR^AGO^, release of N7.45 from C6.47 permits N7.45 to free N7.49 side-chain (of the NPxxY motif) from N7.44, enabling the interaction between N7.49 and D2.50 (Figure 3A). Stability of D2.50 is reinforced by conformation changes of residue S3.39 from *g+* (pointing towards TM3’s backbone in β_2_AR^INV^) to *trans* (interacting with D2.50 in β_2_AR^AGO^). Unsurprisingly, S3.39 is highly conserved (71%) in class A GPCRs. Thus, C6.47-N7.45 interaction keeps N7.49 away from D2.50 and disruption of this hydrogen bond favors N7.49-D2.50 interaction.

In bRho^AGO^, T7.44(*t*) allows Y7.48 to interact with the backbone carbonyl of 7.41. The displacement of Y7.48 away from N7.49 allows the latter to interact with D2.50 through a water molecule resolved in the crystal structure. On the other hand, interaction C6.47-T7.44 in bRho^INV^ disables N7.49-D2.50 interaction and retains Y7.48 close to N7.49 (Figure 3B). Unsurprisingly, Y7.48 is 80% conserved among opsins, backing the importance of this residue within this subfamily. Y7.48 could not populate the conformation found in the crystal if C6.47 would be in the *g+* rotamer. This explains that C6.47 is found in the *trans* rotamer. On the other hand, sRho features H7.48 (relatively rare in opsins; 7%). Given the smaller side chain of His compared to Tyr, residue C6.47 can populate the *g+* rotamer in the former. Still, Q6.43 would prevent N7.49 from interacting with D2.50.

Together with Y7.53, N7.49 is described to undergo a conformational change that helps the release of R3.50 from D/E3.49 (and from D/E6.30 in one third of the receptors). This permits R3.50 to adopt an extended conformation that favors the interaction with the C-terminal portion of Gα domain of G-proteins [64-66]. These conformational changes open a cavity between TMs 3, 5 and 6, allowing G-protein binding [12]. In the context of the global toggle switch model proposed by Schwartz and collaborators [30], where various non-covalent interactions form an allosteric interface that connects ligand binding with intracellular signaling, C(S,T)6.47 could be considered as an intermediate micro-switch modulating interaction between N7.45 and N7.49, thus ultimately modulating TM2-TM7 interaction via N7.49 and D2.50. In the cascade of events during GPCR activation, C6.47 micro-switch would be preceding N7.49.

The analysis of hydrogen bond networks associated to position 6.47 reveals that receptors having C(S,T)6.47 in *gauche*+/- conformation rarely have hydrogen-bond capable residue at position 7.44 (see Table 2). In this situation, C(S,T)6.47 forms hydrogen bond with the side chain of residue at position 7.45. On the other hand, in those structures where residue 6.47 is in the *trans* rotamer conformation, position 7.45 contains Ser (too short to permit a 6.47-7.45 hydrogen bond). In such cases C(S,T)6.47 forms a hydrogen bond with position 7.44 instead. Even with differences at position 6.47 in rotamer conformation (*t*/*g*+/*g*-) and, to a lesser extent, in residue occurrence (Cys/Ser/Thr), all crystal structures are in accordance with a hydrogen bond network that connects residue at 6.47 with N7.49 of the NPxxY motif and the highly conserved (93%) D2.50, via N7.45(or S7.44).

## Conclusions

The largely conserved Cys6.47 of the CWxP motif of class-A GPCRs was initially thought to modulate the conformation of the TM6 proline-kink through a rotamer switch during the process of activation. The release of several agonist-bound (or active-like) crystal structures invalidated this possibility, as no conformational change of this residue has been observed, leaving the role and the prevalence of Cys6.47 unexplained. The present analysis of crystal structures suggests that C6.47 has an active role in rearranging the TM6/7 interface between active and inactive states. Inactive structures are characterized by an interaction between the side chains of residues C(S/T)6.47 and 7.44/7.45, and this generates a constraint in the TM6-TM7 interface that keeps N7.49 (of the NPxxY motif) away from D2.50. Because active structures do not exhibit the interaction between 7.44/7.45 and 6.47, the formation of a hydrogen bond between N7.49 and D2.50 is enabled. We propose that high conservation of Cys at this position is attributable to the requirement of a small residue with ability to form both intra- and extra- main chain hydrogen bonds without altering TM6 Pro-kink. Cys has specific rotameric preferences compared to Ser or Thr, and in addition, the thiol group forms less directional hydrogen bonds than the hydroxyl group.

Although none of the different approaches presented in this study corroborates *per se* our hypothesis, their combination brings to a unique possible role for Cys6.47 in class A GPCRs: Cys6.47 is the gate keeper of the hydrogen bond network involving N7.45, N.7.49 and D2.50, one of the most important micro-switches associated to GPCR activation.

## Methods

### Molecular dynamics simulations of model peptides

Four 25-residue long α-helical polyalanine peptides containing variants of the XAAP (being X = A, C, S and T) motif at their centers were built using PyMol [67]. Each peptide was subsequently embedded into an equilibrated cyclohexane box, to mimic membrane environment. Ten systems were generated by selecting all possible χ_1_ conformers (*gauche+, gauche-* and *trans*) of residue X. All simulations were performed with the GROMACS 4.0.7 simulation package [68]. The force field for the amino acids was the ported version of Amber99sb for this simulation package [69]. Cyclohexane parameters were taken from the methylene groups of Berger’s lipids [70] as employed elsewhere [71]. Systems were energy minimized and subsequently subjected to 50 ns MD simulations. Peptides were restrained to its original conformation during the first 5 ns. Additional 20 ns were discarded as equilibration and the analysis was performed on the last 25 ns. The system was kept at an isotropic pressure of 0.1 MPa using a Berendsen barostat [72]. Temperature was maintained constant at 300 K using separate V-rescale thermostats for the peptide and for cyclohexane [73]. All bonds and angles were frozen using the LINCS algorithm [74]. Lennard-Jones interactions were computed using a cutoff of 1.0 nm and the electrostatic interactions were treated using PME with the same real-space cutoff. Integration of equations of motion was performed using a time-step of 2 fs.

For each XAAP peptide, Φ and Ψ backbone dihedrals and unit bend and unit twist angles within residues *i-7* to *i + 4* (with position *i* containing X = C/S/T/A) were computed. GROMACS tools and HELANAL program [75] were used for this purpose. Unit twist angle is a useful measure of local helix distortion interpreted as follows: an ideal α-helix, with approximately 3.6 residues per turn, has a twist angle of approximately 100° (360°/3.6). Accordingly, a tighter helical segment contains <3.6 and exhibits a twist >100°, whereas a wider segment contains >3.6 residues per turn, resulting in a twist <100°.

### Analysis of sequence positions

Frequencies of aminoacids at a specific position were extracted from GPCRs Motif Searcher (http://lmc.uab.cat/gmos). All data shown herein corresponds to a sequence alignment for all human non-olfactory class A GPCRs.

## Competing interests

The authors declare that they have no competing interests.

## Authors’ contributions

MO and AC carried out the molecular dynamic simulations of model peptides and did the analysis of trajectories. All authors participated in the analysis of crystal structures, development of hypothesis and writing of the manuscript. All authors read and approved the final manuscript.

## Supplementary Material

Additional file 1: Figure S1 Analysis of TM6 irregularities computed from MD simulations of polyalanine peptides containing the CWAP, SWAP, TWAP motifs in the gauche-, *gauche+* and trans conformers. Difference between average Φ (A) and Ψ (B) dihedral angles, unit bend (C) and unit twist (D) profiles. Bend and twist angles assigned at position i correspond to the value computed for (*i-3, i*) and (*i, i+3*) and for (*i-3, i*), respectively. Click here for file
