# Supplementary figures and images for: The role of Cysteine 6.47 in class A GPCRs
Source: BMC Struct Biol. 2013 Mar 15;13:3. doi: 10.1186/1472-6807-13-3 (PMC3610275; doi:10.1186/1472-6807-13-3)

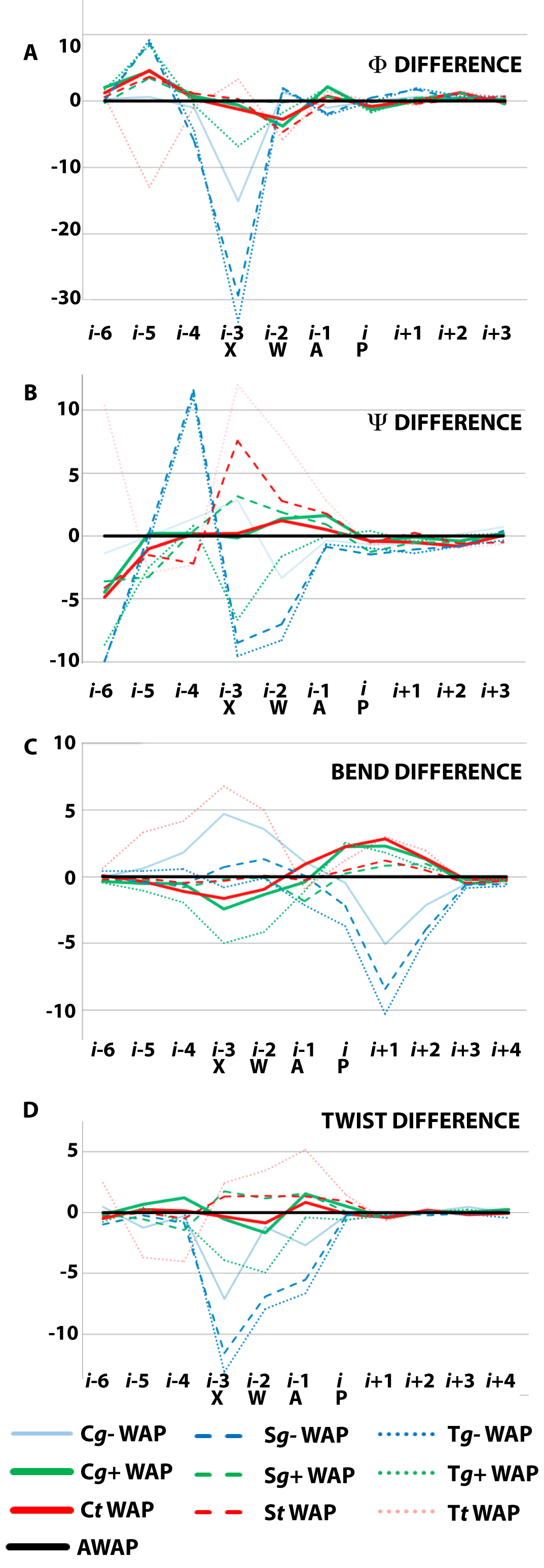

Supplement: Additional file 1: Figure S1 — Analysis of TM6 irregularities computed from MD simulations of polyalanine peptides containing the CWAP, SWAP, TWAP motifs in the gauche-, gauche+ and trans conformers. Difference between average Φ (A) and Ψ (B) dihedral angles, unit bend (C) and unit twist (D) profiles. Bend and twist angles assigned at position i correspond to the value computed for (i-3, i) and (i, i+3) and for (i-3, i), respectively. [file 1472-6807-13-3-S1.tiff]
